# Early nutritional programming in sterlet sturgeon (*Acipenser ruthenus*) with dietary soybean meal: Assessment of growth performance, body composition, and expression of *GH*, *IGF-I*, and *Ghrelin* genes

**DOI:** 10.1371/journal.pone.0299203

**Published:** 2024-03-14

**Authors:** Faezeh Mortezaei, Bahram Falahatkar, Mir Masoud Sajjadi, Roghieh Safari

**Affiliations:** 1 Fisheries Department, Faculty of Natural Resources, University of Guilan, Sowmeh Sara, Guilan, Iran; 2 Department of Fisheries, Gorgan University of Agricultural Sciences and Natural Resources, Gorgan, Iran; Tanta University Faculty of Agriculture, EGYPT

## Abstract

This study was performed to assess the impacts of introducing diets containing different levels of soybean meal (SBM) to sterlet sturgeon (*Acipenser ruthenus*) larvae on growth performance, body composition, and molecular responses in the juvenile stage. The sterlet larvae (57.68 ± 0.66 mg) were weaned onto the formulated diets as follows: a control diet containing 60% fishmeal (FM), and three experimental diets with replacement levels of 15% (SBM15), 30% (SBM30), and 45% (SBM45) of FM with SBM. Then, a total of 260 fish (initial weight: 323.33 ± 11.76 mg) were fed the four different diets for 28 days in triplicates (phase 1, nutritional programming, NP). All treatments were then fed with the FM diet in phase 2 (common phase), and in phase 3 (challenge phase), all experimental groups (6.14 ± 0.08 g) were transitioned to SBM45 for 28 days. At the end of phases 1 and 2, growth performance showed no significant differences among the groups (*P* > 0.05), while significantly improved in SBM45 than the control at the end of phase 3 (*P* < 0.05). No significant differences were found among the groups in any phases for whole body composition (*P* > 0.05). Additionally, the total saturated fatty acids were significantly higher in SBM-based diets than FM at the end of phase 3 (*P* < 0.05). The mRNA of *GH*, *IGF-I* was significantly affected by variation of FM replacement level (*P* < 0.05). The expression level of *Ghrelin* was up-regulated in fish fed SBM at the end of phase 3 (*P* < 0.05). Our findings revealed that NP can positively enhance the adaptation of juvenile sterlet sturgeon to 45% SBM when exposed to the same diets at the larval stage. Further research is being carried out to provide valuable insights into the underlying mechanisms of digestive performance for this species.

## Introduction

The development of socio-economic status in the world relies on food security. Aquaculture plays a critical role in overcoming this issue by providing the fastest-growing animal production and contributing significantly to human protein consumption worldwide [1]. It is most likely that intensive systems will be the best way to meet increasing market demand in aquaculture facilities, and highly dependent on a sufficient supply of high-quality feed at reasonable costs [2, 3].

Fishmeal (FM) has been the most important and widely preferred protein ingredient for the aquaculture feed industry due to its high digestible energy, well-balanced amino acid profile, and bioactive compounds [4, 5]. However, the elevated levels of FM have led to increased feed costs, which are disadvantageous to aquaculturists and have become unsustainable in the long term [6]. Accordingly, the efficient use of high-quality products is required for the growth of sustainable aquaculture. Most of the studies demonstrated that alternative ingredients, such as plant origin, can be used to reduce the amount of FM in traditional feed formulations [5, 7].

In aquafeeds, soybean meal (SBM) provides the best potential source of protein partial or total replacement for FM based on high protein content, well-balanced amino acid profile, and very low costs compared to FM [8, 9]. Over the last decade, a considerable number of nutritional studies have focused on the effects of SBM on fish physiology, showing that high levels of replacement may have negative impacts on fish growth, health, welfare, or disease resistance, due to anti-nutritional factors (ANFs) including lectins, saponins, phytic acid and protease inhibitors amongst others [10–13]. These ANFs can reduce feed intake and nutrient utilization of plant-based diets and affect the bioavailability of amino acids, minerals, or vitamins [14]. Moreover, the exclusion of ANFs from plant-based diets through further processing such as chemical or heat treatments requires additional costs that are economically unfeasible [15]. Nowadays, some studies have focused on employing an alternative strategy to adapt fish to a plant-based diet rather than trying to alter the diets of the fish, which is termed nutritional programming (NP). These studies reinforce the idea that, as a result of adaptive changes at cellular, molecular, and biochemical levels, nutritional changes during critical periods of early development may permanently induce changes in animal metabolism and physiology [16–18].

Some prospective studies have shown a positive link between NP at the early stage of life, and growth, feed utilization, protein, lipids, and glucose metabolism in late life, suggesting that an epigenetic component, such as DNA methylation or histone modification, which can preserve a permanent memory of previous nutritional periods, may be present in developmental stages [18–20]. However, NP studies have just been started, and there are still unanswered questions regarding causes, results, persistence, and underlying mechanisms.

One of the intensively developing fields in fish production is sturgeon aquaculture. The market price, rich nutritional qualities of meat and caviar, rapid growth, and adaptability to changes in the natural environment are considered crucial factors that make sturgeon species the most valuable fish in commercial aquaculture [21, 22]. However, intensive aquaculture production of sturgeon has proved to be challenging, particularly at early development stages because of a high mortality rate. Hence, recent studies have focused on the development of optimal larval-rearing methods to improve survival rate and physiological performance [23–25].

Sterlet sturgeon *Acipenser ruthenus*, an important species of sturgeon for farming in Eastern and Central Europe, has been of great attention to fish farmers, and consumers, attributable to the delightful taste of meat and caviar [26, 27]. Sterlet has been also known as a model in aquaculture research including physiology and nutrition, due to its rapid growth rate and the environmental requirements of freshwater [28–30]. Moreover, it is a relative carnivore species using a high concentration of protein (48–52%) in the larval stage to achieve optimal growth and market size serving as an appropriate model for studying plant protein utilization and innovative feeding strategies [31, 32].

NP has provided new insights into the determination of metabolic processes and molecular pathways affected by the substitution of FM with plant-based ingredients in fish nutrition, but research has been conducted on only a small number of fish species, and mechanisms underlying such changes are currently unknown. Furthermore, the importance of larval endogenous feeding should not be overlooked, due to the high degree of vulnerability to any nutritional stimuli. So, detailed knowledge of larval and juvenile nutritional requirements is key importance for the success of such optimum rearing strategies [33, 34]. The development of nutritionally balanced feeds that meet the nutrient requirements and applying the appropriate nutritional strategies for the optimal growth of sterlet sturgeon is necessary for making aquaculture sustainable and profitable, as feed contributes the most to the operational cost [35, 36]. Therefore, the hypothesis behind this study was that early feeding of sterlet sturgeon with the plant-based diet stimuli could enhance the physiological adaptation of the fish to the same diets, at future life stages, and the specific objectives of this study were to examine the growth or feeding performance, body composition, and growth and appetite-related gene expression of juvenile sterlet sturgeon, when experienced the SBM-based diets with different levels of FM substitution during exogenous feeding.

## Materials and methods

### Ethical approval for animal use

All experimental methods were performed in accordance with the recommendations to reduce stress and maintain fish welfare, which were approved by the Committee of Ethics and Animal Experimentation of University of Guilan, Iran (IR.GUILAN.REC.1401.089).

### Diets preparation

Four experimental diets were formulated to be isonitrogenous (48% crude protein) and isoenergetic (20 KJ g^-1^): a diet including FM as a main protein source was considered as the control diet (FM) and in three other diets, soybean meal replaced 15% (SBM15), 30% (SBM30) and 45% (SBM45) of FM (Table 1). After the formulation, the experimental diets were produced by Abzigostar Avan Feed Manufacturer (Behshahr, Iran). All dry ingredients were micronized to 0.3 mm by a pulverizer mill (HM Series 38, CPM, California, US) and then, vegetable and fish oils with soy lecithin were added to the dry ingredients and mixed for 15 min. After adding micro-ingredients and water (~10% of the total mass of feed), diets were pelleted and grounded again. The pellets for different trials were sieved to appropriate sizes (0.5–1 mm in diameter) and stored at -20˚C until performing the later experiments. Chemical analysis was conducted according to AOAC methods [37] to determine crude protein, crude fat, ash, and moisture contents.

**Table 1:** Formulation and proximate analysis of experimental diets (% dry matter).

Ingredients	Experimental diets			
	FM	SBM15	SBM30	SBM45
Fishmeal^a^	60.00	51.00	42.00	33.00
Soybean meal^b^	0.00	9.00	18.00	27.00
Wheat gluten^b^	1.50	3.00	5.50	8.80
Wheat bran^b^	7.00	6.40	6.80	4.00
Soy fiber^b^	2.50	2.90	1.90	1.00
Corn flour^b^	4.70	4.70	4.10	5.50
Wheat flour^b^	9.00	9.40	7.00	4.30
Soy lecithin^c^	4.30	3.00	3.60	4.00
Fish oil^a^	2.30	2.10	2.35	3.00
Linseed oil^d^	2.30	2.10	2.35	3.00
Sodium alginate^e^	1.00	1.00	1.00	1.00
Vitamin C^f^	0.10	0.10	0.10	0.10
Vitamin premix^g^	1.50	1.50	1.50	1.50
Mineral premix^g^	1.40	1.40	1.40	1.40
Mono calcium phosphate^f^	0.10	0.10	0.10	0.10
Methionine^f^	1.00	1.00	1.00	1.00
Lysine^f^	1.00	1.00	1.00	1.00
Choline chloride^f^	0.10	0.10	0.10	0.10
Sodium chloride	0.10	0.10	0.10	0.10
Toxin binder^f^	0.10	0.10	0.10	0.10
Proximate analysis (%)				
Protein	48.96	48.38	48.49	48.38
Lipid	18.00	17.31	16.80	16.00
Ash	10.13	11.60	10.30	11.40
Moisture	6.50	5.80	6.10	6.50
Gross energy (kJ g^-1^)^h^	20.95	20.12	20.02	20.04

^a^ Anchovy Kilka fishmeal (Tehran Kilka Powder, Sari, Iran)

^b^ Iran’s Government Trading Corporation, Amirabad Port, Behshahr, Iran

^c^ Pacific Commodities Mumbai, India

^d^ Iran’s Government Trading Corporation, Amirabad Port, Behshahr, Iran

^e^ Merck, Darmstadt, Germany

^f^ DSM, Jalisco, México

^g^ Abzigostar Avan Company (Behshahr, Iran)

^h^ Gross energy (GE) was calculated by the formula as follows [38]

GE (KJ g^-1^) = (23.6 × % protein) + (39.5 × % lipid) + (17.2 × % carbohydrate)

### Experimental conditions and rearing phases

#### Broodstock spawning and larval rearing

Feeding trials were carried out at the Dr. Yousefpour Marine Fishes Restocking and Genetic Conservation Center (Siahkal, Guilan, Iran) using a flow-through aquaculture system supplied by Khararoud River. Sterlet broodstock were selected whose oocytes were undergoing polarization and inspected for gonadal development by biopsy. Then, they received an intramuscular injection of 4 μg per kg body weight of LHRHa2 at two intervals [30]. After 24–48 h, they were artificially reproduced, and fertilized eggs were incubated in Yushchenko incubators. During 4–6 days after incubation, larvae were hatched and prepared to transfer into the circular concrete tanks (180 cm diameter, 15 cm depth, 424 L volume).

Seven to nine days post-hatching (dph), larvae were fed eight times a day with newly hatched Artemia nauplii (*Artemia franciscana*) and fine-sieved Daphnia (*Daphnia magna*) at 60–80% of body weight per day [39]. At 13 dph, five hundred and fifty sterlet larvae (Initial weight: 57.68 ± 0.66 mg) were randomly stocked into 12 circular tanks (180 cm diameter, 20 cm depth, 565 L volume), and fed to satiation 6–8 times daily (9:00 AM to 6:30 PM) with frozen Chironomidae larvae (CH). In the adaptation phase, a period of 10 days of co-feeding was performed to adapt the larvae to the formulated diets. At this period (22–32 dph), fish were fed with a 20% daily reduction of CH and stepwise increasing the ratio of the experimental formulated diets (FM, SBM15, SBM30, and SBM45). At 32 dph, CH was completely replaced by the formulated feeds in all feeding trials.

#### Experimental design

The experimental design was carried out over three phases and fish were fed in triplicate. Phase 1 was initiated only after the adaptation. For this purpose, a total of 260 sterlet fry (Initial weight: 323.33 ± 11.76 mg) were randomly restocked into the same tanks and nutritionally programmed (NP) for four weeks by feeding the four diets (FM, SBM15, SBM30, and SBM45. In phase 2, all groups were transitioned to the control diet (FM) and in phase 3, fish were nutritionally challenged for four weeks with diet including 45% of FM replacement (SBM45). The feeding rate was adjusted every 14 days by weighing tank biomass. The feeds were given at a rate of 10% fish biomass and then adjusted to the satiation. Every morning, 20% of the water in the tanks was siphoned to remove any uneaten feed and debris.

During adaptation to the end of phase 3, the average water flow rate was 3.25 ± 0.48 L min^-1^, and the photoperiod consisted of 12L:12D. The average water temperature, pH, and oxygen level were 23.40 ± 2.30°C, 7.88 ± 0.13 and 8.00 ± 0.41 mg L^-1^, respectively.

### Growth performance and feed efficiency

At the end of each phase, feeding was ceased for 24 h, and entire fish from each tank were assessed to determine weight gain (WG), specific growth rate (SGR), body weight increases (BWI), feed efficiency ratio (FER), CF (condition factor), protein efficiency ratio (PER), lipid efficiency ratio (LER), protein productive value (PPV), lipid productive value (LPV), hepatosomatic index (HSI), viscerosomatic index (VSI), cannibalism rate (CR) and survival rate (SR) by following formulas:

WG (g) = final weight (g)—initial weight (g)

SGR (% day^-1^) = 100 × [Ln final weight (g)—Ln initial weight (g) /days]

BWI (%) = 100 × [(WG (g) / initial weight (g)]

FER (%) = 100× [(WG / dry feed intake)]

CF = 100 × [body weight (g) / total length^3^ (cm)]

PER = WG / protein intake

LER = WG / lipid intake

PPV (%) = 100 × [total protein gain (g) / total protein intake (g)]

LPV (%) = 100 × [total lipid gain (g) / total lipid intake (g)]

HSI (%) = 100 × [liver weight (g) / whole fish weight (g)]

VSI (%) = 100 × [viscera weight (g) / whole fish weight (g)]

CR (%) = 100 × [(number of fish at initial—total dead fish removed from the tanks—number of fish at final) / number of fish at initial]

SR (%) = 100 × [number of fish at final / number of fish at initial]

### Chemical analyses

At the end of phases 1–3, 24-h starved whole fish were randomly sampled and stored at −20°C for determination of carcass proximate composition according to AOAC standard procedures [37]. Crude protein content was measured using the Kjeldahl method (V40, Bakhshi, Tehran, Iran) and crude fat content was evaluated by Soxhlet lipid extraction (6XL, Bakhshi, Tehran, Iran). Moisture contents were measured after drying the samples in an oven at 105°C until a constant weight was obtained and ash content was estimated after combustion in an electric furnace (ATRA AFE1200L-3DH, Tehran, Iran) at 550°C for 8 h.

### Fatty acid profile

At the end of phase 3, samples of sterlet juvenile from each replicate were collected and stored at -80°C. Lipids were initially extracted by adding methanol and chloroform (1:1 (v v^-1^)) to determine the total fatty acid content. Afterward, sodium hydroxide methanol/boron-trifluoride/n-hexane (5:3:1) mixture was added as developing solvents to separate the fatty acid methyl esters (FAME) into triglycerides and phospholipids. The esterified samples were then added to gas liquid chromatography (BPX70; Philips, Netherlands) that was outfitted with a flame ionization detector (FID) and SGE capillary column for identification. The capillary column’s temperature was set to 160°C initially then gradually increased to 230°C. The proportion of the total content was used to express the relative amount of fatty acid in each replicate [40, 41].

### Molecular assessments

To identify the molecular changes in the nutritional programmed juvenile sterlet, the growth and appetite-related genes expression were investigated at the end of phases 1 and 3. Fish were kept starved for 24 h, and euthanized by a high dose of clove powder extract. Afterward, the liver, brain, and intestine from each tank were dissected on ice (five fish per replicate), and the samples were assigned to a corresponding tube and immediately snap-frozen in liquid nitrogen. Then, the samples were stored at –80°C until RNA extraction and other analyses. The tissues were homogenized and total RNA extraction and the subsequent cDNA synthesis were conducted using Sinaclone RNX plus Kit (Sinaclone, Tehran, Iran) and GeNet Bio cDNA® synthesis kit (Daejeon, South Korea) according to the manufacturer’s instructions, respectively. The ratio of 260/280 was evaluated to determine the purity of RNA samples using the NanoDrop Spectrophotometer (Nanodrop Technologies, Wilmington, DE, US). The primers for the amplification of growth genes (*IGF-I* and *GH*) were adopted from previous study by Safari et al. [42], and the primer targeting ghrelin (*Ghrelin*) was designed based on the cDNA sequences of these genes in *A*. *ruthenus* available in GenBank database using the Oligo Primer Analysis Software (Ver. 7.60, Colorado, US), which is illustrated in Table 2. Primer efficiency was tested with serial dilutions of a cDNA pool.

**Table 2:** Primer sequences used for quantitative real-time PCR in sterlet sturgeon (*Acipenser ruthenus*).

Primers name	Primer sequences	Description	Tm (°C)	Accession number
*GH*	F:TTCATGATGAGTGCTCCGTTC	Growth	58	AB517597.1
	R:GTCAGAATTCAAGTGGCGAATC			
*IGF-I*	F: CAAACATGATCTGTATGTG	Growth	58	AB512770.1
	R: AGAATTCAAGTGGCGACATG			
*Ghrelin*	F: CGCTTGGGACAGAACATGTA	Appetite	58	MG79214401
	R: ATTCTGACATTGAGATCCCG			
*β- actin*	F: TTGCCATCCAGGCTGTGCT	Housekeeping gene	58	AY878120.1
	R: TCTCGGCTGTGGTGAA			

Real-time quantitative PCR (qPCR) was performed in StepOne Plus real-time PCR instrument (Thermo Fisher Scientific, Wilmington, US) using *β-actin* as the housekeeping gene in a final volume of 10 μl (8 μl master mix and 2 μl cDNA template) per each reaction well in triplicate. The master mix contained 1.5 μl H2O, 0.5 μl of each primer, 0.5 μl of DMSO and 5 μl of the SYBR Green mix (Pars Tous Company, Mashhad, Iran). The PCR conditions were the following: 95°C for 5 min followed by 40 cycles of 95°C for 15 s, 58°C for 30 s, 72°C for 30 s and 95°C for 15 s. Melting-curve analysis was conducted and amplification of a single product was confirmed after each run. The fold expression of each gene is determined by the CT method (2^-ΔΔCT^).

### Statistical analyses

The results were exhibited as means ± standard error (SE). Before the analysis of data, the homogeneity of variances was tested using Levene’s test. At the end of each phase of the experiment, data on growth, proximate composition, and genes expression were statistically subjected to One-way ANOVA using SPSS (ver.16, SPSS Inc., Chicago, US). Additionally, an Analysis of Covariance (ANCOVA) was used to assess whether growth and feeding indices at the onset of nutritional phases had independent effects on the same indices at the end of each phase. The p-value of less than 0.05 was considered as significance level and a Tukey’s test was employed for analyzing the significant differences among treatments.

## Results

### Growth performance during three phases

The results of adaptation phase are shown in Table 3. No significant differences were observed among the treatments for growth indices; however, SR was significantly lower in SBM45 than the other treatments. The highest CR was also detected in SBM45 (*P* < 0.05). At the end of phase 1, there were no significant differences among the groups (Table 4). At the end of phase 2, the final weight was observed slightly higher in FM group, but not significant, and FER was significantly higher in FM than SBM groups. No significant differences were found among the other growth indices (Table 5). The results of growth performance at the end of challenge phase indicated that final weight was significantly higher in SBM45 and significantly lower in FM. Other indices including WG, SGR and BWI significantly differed among the treatments, and the highest values were appeared in SBM45 (Table 6).

**Table 3:** Growth performance of sterlet sturgeon (*Acipenser ruthenus*) larvae fed different levels of soybean meal at the end of the adaptation phase (means ± SE; n = 3).

Indices	Experimental groups				*P* value
	FM	SBM15	SBM30	SBM45	
Initial weight (mg)	58.00 ± 0.58	57.67 ± 1.45	58.33 ± 1.20	56.67 ± 2.19	0.87
Final weight (mg)	283.33 ± 8.82	256.67 ± 21.86	243.33 ± 14.53	240.00 ± 11.54	0.24
Initial length (mm)	21.43 ± 0.26	21.27 ± 0.43	20.90 ± 0.44	21.20 ± 0.21	0.75
Final length (mm)	37.02 ± 0.02	37.49 ± 0.64	35.48 ± 0.77	35.24 ± 1.44	0.20
Weight gain (mg)	225.33 ± 8.65	199.00 ± 21.93	185.00 ± 13.43	183.33 ± 13.35	0.32
Specific growth rate (% day^-1^)	14.44 ± 0.20	13.49 ± 0.72	13.25 ± 0.24	13.05 ± 0.80	0.36
Body weight increase (%)	389.61 ± 11.20	343.46 ± 34.85	329.74 ± 11.33	323.24 ± 36.24	0.34
Condition factor	0.55 ± 0.02	0.48 ± 0.03	0.57 ± 0.04	0.54 ± 0.02	0.29
Feed efficiency ratio (%)	52.83 ± 1.66	46.24 ± 4.62	44.96 ± 1.75	42.49 ± 3.55	0.20
Cannibalism rate (%)	4.00 ± 0.58^b^	5.13 ± 0.41^ab^	5.90 ± 0.67^ab^	8.33 ± 1.45^a^	0.04
Survival rate (%)	75.33 ± 0.01^a^	74.33 ± 0.01^a^	73.00 ± 0.01^ab^	67.67 ± 0.02^b^	0.02

Lower case letters in each row denote significant differences between the experimental groups. FM: fishmeal as the main protein source; SBM15: SBM replacing 15% of FM; SBM30: SBM replacing 30% of FM; SBM45: SBM replacing 45% of FM.

**Table 4:** Growth performance of sterlet sturgeon (*Acipenser ruthenus*) larvae fed different levels of soybean meal at the end of programming phase (means ± SE; n = 3).

Indices	Experimental groups				*P* value
	FM	SBM15	SBM30	SBM45	
Initial weight (mg)	306.67 ± 24.04	306.67 ± 8.82	323.33 ± 12.01	256.67 ± 37.56	0.44
Final weight (g)	1.52 ± 0.03	1.44 ± 0.04	1.44 ± 0.06	1.46 ± 0.05	0.48
Initial length (cm)	3.59 ± 1.46	3.81 ± 0.64	3.61 ± 0.78	3.77 ± 0.51	0.21
Final length (cm)	6.91 ± 0.20	6.94 ± 0.09	6.86 ± 0.12	6.76 ± 0.09	0.80
Weight gain (g)	1.21 ± 0.03	1.13 ± 0.05	1.11 ± 0.05	1.11 ± 0.04	0.33
Specific growth rate (% day^-1^)	5.87 ± 0.24	5.56 ± 0.19	5.29 ± 0.16	5.08 ± 0.31	0.17
Body weight increase (%)	398.30 ± 40.08	375.21 ± 25.42	340.55 ± 18.98	318.23 ± 37.35	0.35
Condition factor	0.47 ± 0.04	0.44 ± 0.01	0.45 ± 0.04	0.47 ± 0.03	0.84
Feed efficiency ratio (%)	59.40 ± 1.81	51.51 ± 7.61	51.27 ± 6.18	48.66 ± 2.10	0.51
Lipid efficiency ratio	3.49 ± 0.11	3.30 ± 0.49	3.23 ± 0.39	2.98 ± 0.13	0.74
Lipid productive value (%)	4.59 ± 0.14	3.91 ± 0.79	4.37 ± 0.19	3.45 ± 0.54	0.42
Protein productive value (%)	6.01 ± 0.15	6.45 ± 0.80	5.78 ± 0.39	6.23 ± 0.68	0.86
Hepatosomatic index (%)	1.04 ± 0.48	2.40 ± 0.33	2.15 ± 1.02	1.25 ± 0.28	0.36
Viscerosomatic index (%)	4.39 ± 0.82	5.98 ± 0.86	3.97 ± 2.03	3.82 ± 0.91	0.63
Survival rate (%)	85.77 ± 2.12	80.64 ± 5.42	83.08 ± 4.28	79.11 ± 6.22	0.78

FM: fishmeal as the main protein source; SBM15: SBM replacing 15% of FM; SBM30: SBM replacing 30% of FM; SBM45: SBM replacing 45% of FM.

**Table 5:** Growth performance of sterlet sturgeon (*Acipenser ruthenus*) fed different levels of soybean meal at the end of phase 2 (means ± SE; n = 3).

Indices	Experimental groups				*P* value
	FM	SBM15	SBM30	SBM45	
Initial weight (g)	1.52 ± 0.03	1.44 ± 0.04	1.44 ± 0.06	1.46 ± 0.05	0.48
Final weight (g)	6.29 ± 0.06	6.27 ± 0.18	5.80 ± 0.17	6.19 ± 0.07	0.09
Initial length (cm)	6.91 ± 0.20	6.94 ± 0.09	6.86 ± 0.12	6.76 ± 0.09	0.80
Final length (cm)	11.01 ± 0.07	11.01 ± 0.21	10.87 ± 0.08	10.76 ± 0.15	0.56
Weight gain (g)	4.77 ± 0.06	4.85 ± 0.18	4.37 ± 0.22	4.72 ± 0.11	0.22
Specific growth rate (% day^-1^)	5.07 ± 0.07	5.28 ± 0.13	5.00 ± 0.23	5.16 ± 0.15	0.66
Body weight increase (%)	313.72 ± 7.80	338.59 ± 16.38	307.66 ± 25.62	324.13 ± 17.98	0.66
Condition factor	0.47 ± 0.01	0.47 ± 0.04	0.45 ± 0.01	0.50 ± 0.03	0.64
Feed efficiency ratio (%)	110.83 ± 1.07^a^	99.45 ± 0.89^b^	100.38 ± 0.90^b^	94.97 ± 0.81^c^	0.00
Lipid efficiency ratio	6.83 ± 0.22	6.53 ± 0.18	6.23 ± 0.06	6.54 ± 0.48	0.68
Lipid productive value (%)	2.69 ± 0.22	2.57 ± 0.12	3.02 ± 0.21	2.59 ± 0.09	0.29
Protein productive value (%)	4.26 ± 0.20	3.68 ± 0.08	4.29 ± 0.21	4.31 ± 0.07	0.51
Hepatosomatic index (%)	2.24 ± 0.76	2.38 ± 0.39	1.61 ± 0.36	2.16 ± 0.28	0.70
Viscerosomatic index (%)	9.97 ± 0.67	9.09 ± 0.29	9.10 ± 0.22	8.92 ± 0.69	0.37
Survival rate (%)	97.51 ± 0.86	96.59 ± 1.40	98.23 ± 0.56	97.97 ± 0.19	0.58

Lower case letters in each row denote significant differences between the experimental groups. FM: fishmeal as the main protein source; SBM15: SBM replacing 15% of FM; SBM30: SBM replacing 30% of FM; SBM45: SBM replacing 45% of FM.

**Table 6:** Growth performance of sterlet sturgeon (*Acipenser ruthenus*) fed different levels of soybean meal at the end of challenge phase (means ± SE; n = 3).

Indices	Experimental groups				*P* value
	FM	SBM15	SBM30	SBM45	
Initial weight (g)	6.29 ± 0.06	6.27 ± 0.18	5.80 ± 0.17	6.19 ± 0.07	0.09
Final weight (g)	16.16 ± 0.38^b^	16.91 ± 0.14^ab^	16.70 ± 0.16^ab^	17.54 ± 0.30^a^	0.04
Initial length (cm)	11.01 ± 0.07	11.01 ± 0.21	10.87 ± 0.08	10.76 ± 0.15	0.56
Final length (cm)	15.52 ± 0.11	15.88 ± 0.04	15.81 ± 0.07	15.88 ± 0.28	0.37
Weight gain (g)	9.87 ± 0.40^b^	10.63 ± 0.84^ab^	10.91 ± 0.10^ab^	11.35 ± 0.28^a^	0.02
Specific growth rate (% day^-1^)	3.37 ± 0.10^b^	3.54 ± 0.08^ab^	3.71 ± 0.02^a^	3.72 ± 0.06^a^	0.03
Body weight increase (%)	156.98 ± 7.00^b^	169.32 ± 5.96^ab^	181.84 ± 1.51^ab^	183.51 ± 4.67^a^	0.02
Condition factor	0.43 ± 0.01	0.42 ± 0.00	0.42 ± 0.01	0.44 ± 0.02	0.53
Feed efficiency ratio (%)	62.45 ± 2.52	64.75 ± 2.36	73.75 ± 2.05	72.71 ± 5.52	0.11
Lipid efficiency ratio	3.67 ± 0.15	4.14 ± 0.15	4.65 ± 0.13	4.45 ± 0.34	0.05
Lipid productive value (%)	1.87 ± 0.08	1.87 ± 0.07	1.92 ± 0.18	1.81 ± 0.10	0.90
Protein productive value (%)	1.58 ± 0.13	1.48 ± 0.07	1.60 ± 0.04	1.58 ± 0.09	0.78
Hepatosomatic index (%)	2.16 ± 0.40	2.04 ± 0.05	2.31 ± 0.29	2.24 ± 0.11	0.89
Viscerosomatic index (%)	7.82 ± 0.58	8.17 ± 0.14	8.30 ± 0.77	7.69 ± 0.23	0.81
Survival rate (%)	99.57 ± 0.21	99.61 ± 0.30	99.47 ± 0.27	99.67 ± 0.19	0.94

Lower case letters in each row denote significant differences between the experimental groups.

FM: fishmeal as the main protein source; SBM15: SBM replacing 15% of FM; SBM30: SBM replacing 30% of FM; SBM45: SBM replacing 45% of FM

### Body composition

Tables 7–9 represent the chemical compositions of sterlet from the phase 1 to the phase 3 during 12 weeks. According to the results, there were no significant differences among the groups at each phase.

**Table 7:** Proximate body composition of sterlet sturgeon (*Acipenser ruthenus*) at the end of programming phase (means ± SE; n = 3).

Proximate analysis	Experimental groups				*P* value
	FM	SBM15	SBM30	SBM45	
Crude protein (%)	6.25 ± 0.08	6.88 ± 0.16	6.06 ± 0.49	6.75 ± 0.40	0.30
Crude fat (%)	1.59 ± 0.04	1.33 ± 0.09	1.53 ± 0.20	1.26 ± 0.12	0.26
Moisture (%)	90.16 ± 0.52	89.34 ± 0.53	89.36 ± 0.17	89.41 ± 0.51	0.55
Ash (%)	2.14 ± 0.14	1.96 ± 0.29	1.84 ± 0.13	2.13 ± 0.15	0.82

FM: fishmeal as the main protein source; SBM15: SBM replacing 15% of FM; SBM30: SBM replacing 30% of FM; SBM45: SBM replacing 45% of FM.

**Table 8:** Proximate body composition of sterlet sturgeon (*Acipenser ruthenus*) at the end of common phase (means ± SE; n = 3).

Proximate analysis	Experimental groups				*P* value
	FM	SBM15	SBM30	SBM45	
Protein (%)	8.62 ± 0.42	8.46 ± 0.32	8.91 ± 0.01	9.44 ± 0.37	0.23
Lipid (%)	2.01 ± 0.04	1.91 ± 0.12	2.07 ± 0.05	1.94 ± 0.06	0.47
Moisture (%)	85.90 ± 0.42	85.86 ± 0.42	85.17 ± 0.06	85.51 ± 0.68	0.47
Ash (%)	2.97 ± 0.54	2.60 ± 0.28	3.75 ± 0.37	2.76 ± 0.08	0.41

FM: fishmeal as the main protein source; SBM15: SBM replacing 15% of FM; SBM30: SBM replacing 30% of FM; SBM45: SBM replacing 45% of FM.

**Table 9:** Proximate body composition of sterlet sturgeon (*Acipenser ruthenus*) at the end of challenge phase (means ± SE; n = 3).

Proximate analysis	Experimental groups				*P* value
	FM	SBM15	SBM30	SBM45	
Protein (%)	12.14 ± 1.05	11.73 ± 0.13	11.38 ± 0.03	11.88 ± 0.23	0.80
Lipid (%)	5.03 ± 0.22	4.82 ± 0.27	4.51 ± 0.35	4.64 ± 0.36	0.67
Moisture (%)	75.96 ± 0.17	75.85 ± 0.43	77.40 ± 0.94	76.52 ± 0.48	0.28
Ash (%)	5.60 ± 0.07	5.19 ± 0.30	5.02 ± 0.41	5.31 ± 0.37	0.62

FM: fishmeal as the main protein source; SBM15: SBM replacing 15% of FM; SBM30: SBM replacing 30% of FM; SBM45: SBM replacing 45% of FM.

### Fatty acids composition

Data for fatty acid profile of juvenile sterlet during phase 3 are presented in Table 10. C14:0 values were significantly lower in FM and SBM15 than the other groups, and there were not significant differences in FAs composition among the experimental groups. Total saturated fatty acids (SFA) were significantly higher in SBM30 than FM and SBM15, but no significant differences were detected in SBM30 and SBM45.

**Table 10:** Fatty acid composition of juvenile sterlet (*Acipenser ruthenus*) at the end of challenge phase (means ± SE; n = 3).

Fatty acids	Experimental groups				*P* value
	FM	SBM15	SBM30	SBM45	
C14:0	0.30 ± 0.01^b^	0.20 ± 0.03^b^	1.11 ± 0.05^a^	1.08 ± 0.01^a^	0.00
C15:0	0.24 ± 0.02	0.18 ± 0.03	0.27 ± 0.07	0.27 ± 0.05	0.46
C16:0	21.23 ± 0.10	21.45 ± 0.15	21.47 ± 0.14	21.03 ± 0.14	0.14
C16:1	5.29 ± 0.07	5.24 ± 0.16	5.32 ± 0.09	5.24 ± 0.01	0.92
C17:0	0.35 ± 0.01	0.34 ± 0.01	0.36 ± 0.02	0.35 ± 0.01	0.72
C17:1	0.44 ± 0.01	0.44 ± 0.01	0.44 ± 0.01	0.47 ± 0.01	0.26
C18:0	3.29 ± 0.05	3.38 ± 0.10	3.31 ± 0.01	3.37 ± 0.09	0.75
C18:1(n-9)C	40.45 ± 0.34	40.63 ± 0.34	39.63 ± 0.38	40.11 ± 0.26	0.23
C18:1(n-11)C	0.11 ± 0.01	0.14 ± 0.01	0.11 ± 0.01	0.13 ± 0.01	0.07
C18:2(n-6)C	16.81 ± 0.12	16.57 ± 0.24	17.11 ± 0.15	17.17 ± 0.13	0.12
C18:3 n6	1.47 ± 0.12	1.53 ± 0.05	1.32 ± 0.05	1.38 ± 0.04	0.25
C18:3 n3	0.88 ± 0.01	0.85 ± 0.04	0.90 ± 0.03	0.90 ± 0.02	0.67
C20:0	0.36 ± 0.15	0.34 ± 0.01	0.33 ± 0.02	0.33 ± 0.01	0.39
C20:1	0.13 ± 0.01	0.12 ± 0.01	0.11 ± 0.01	0.14 ± 0.01	0.20
C20:2	0.95 ± 0.01	0.97 ± 0.03	0.95 ± 0.02	0.97 ± 0.03	0.82
C20:3 n9	0.53 ± 0.02	0.54 ± 0.01	0.51 ± 0.03	0.57 ± 0.01	0.30
C20:3 n6	0.50 ± 0.03	0.47 ± 0.01	0.47 ± 0.03	0.47 ± 0.01	0.72
C20:3 n3	0.93 ± 0.01	0.85 ± 0.04	0.87 ± 0.05	0.93 ± 0.02	0.40
C20:4 n6 ARA	0.05 ± 0.01	0.05 ± 0.01	0.03 ± 0.01	0.03 ± 0.01	0.11
C20:4 n3	0.10 ± 0.01	0.09 ± 0.01	0.17 ± 0.01	0.10 ± 0.01	0.42
C20:5 n3 EPA	0.78 ± 0.04	0.75 ± 0.03	0.78 ± 0.01	0.85 ± 0.05	0.35
C22:0	0.12 ± 0.05	0.17 ± 0.04	0.11 ± 0.03	0.11 ± 0.01	0.59
C22:1	0.13 ± 0.01	0.10 ± 0.02	0.13 ± 0.01	0.14 ± 0.01	0.14
C24:0	0.16 ± 0.01	0.15 ± 0.01	0.15 ± 0.01	0.16 ± 0.01	0.56
C22:4 n6 DTA	0.36 ± 0.01	0.36 ± 0.01	0.35 ± 0.02	0.37 ± 0.02	0.63
C22:5 n6	0.26 ± 0.01	0.23 ± 0.02	0.24 ± 0.01	0.26 ± 0.01	0.13
C22:6 n3 DHA	3.53 ± 0.14	3.24 ± 0.09	3.33 ± 0.20	3.54 ± 0.07	0.38
SFA	26.04 ± 0.17^c^	26.19 ± 0.15^bc^	27.10 ± 0.08^a^	26.71 ± 0.17^ab^	0.00
MUFA	46.55 ± 0.39	46.68 ± 0.22	45.74 ± 0.48	46.22 ± 0.27	0.31
n-3	6.41 ± 0.01	5.78 ± 0.15	6.05 ± 0.24	6.32 ± 0.03	0.05
n-6	19.45 ± 0.09	19.21 ± 0.19	19.51 ± 0.21	19.68 ± 0.09	0.28
n3/n6	0.32 ± 0.03	0.30 ± 0.01	0.31 ± 0.02	0.32 ± 0.01	0.42
EPA/DHA	0.22 ± 0.00	0.23 ± 0.01	0.24 ± 0.01	0.24 ± 0.02	0.73
PUFA	27.46 ± 0.04	27.39 ± 0.08	27.42 ± 0.05	27.54 ± 0.04	0.09
HUFA	7.31 ± 0.08^a^	6.57 ± 0.12^ab^	6.75 ± 0.27^ab^	7.12 ± 0.08^ab^	0.04
ALA/LA	19.04 ± 0.27	19.57 ± 0.80	19.13 ± 0.67	19.17 ± 0.38	0.91

Lower case letters in each row denote significant differences between the experimental groups. FM: fishmeal as the main protein source; SBM15: SBM replacing 15% of FM; SBM30: SBM replacing 30% of FM; SBM45: SBM replacing 45% of FM.

### Molecular analysis

**Fig 1:**
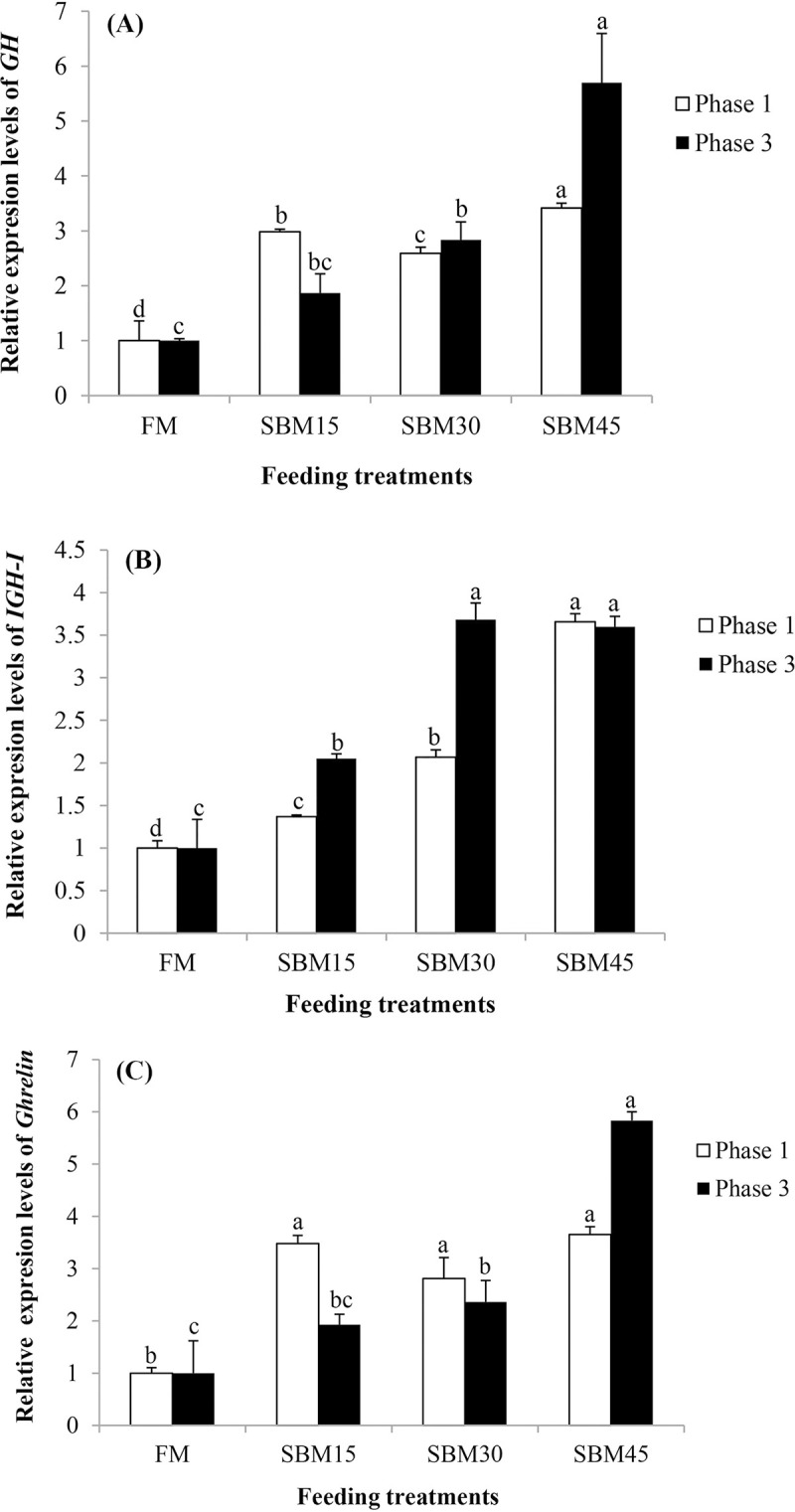
Relative expression of the growth (*GH* and *IGF-I*) and appetite (*Ghrelin*) related genes. mRNA levels of *GH* gene in the brain (A), *IGF-I* gene in the liver (B), and *Ghrelin* gene in the intestine (C) of sterlet sturgeon (*Acipenser ruthenus*) fed on experimental diets at the end of the phases 1 and 3 (n = 3). Means ± SE for the bars with unlike letters are significantly different (*P* < 0.05). FM: Fishmeal as the main protein source; SBM15: Soybean meal replacing 15% of FM; SBM30: SBM replacing 30% of FM; SBM45: SBM replacing 45% of FM.

As shown in Fig 1A, the mRNA level of *GH* was significantly up-regulated in SBM45 group than the other groups whether in phase 1 or 3 (*P* < 0.05). The *GH* relative expression in FM, SBM15 and SBM30 was similar in phase 3 and not significant (*P* > 0.05). An increasing trend was observed in *IGF-I* gene expression with incremental level of soybean inclusion in sterlet diets at the end of programming phase (Fig 1B) and it was significantly down-regulated in FM than the other groups at the end of third phase (*P* < 0.05). The relative mRNA levels of *Ghrelin* gene, which related to appetite is shown in Fig 1C. The gene was significantly up-regulated in intestine of fish fed SBM than FM at programming phase; however, in phase 3, the expression level ‌in SBM45 group was significantly higher than the other groups (*P* < 0.05).

## Discussion

At the end of the adaptation period in sterlet larvae, SR was significantly lower in SBM45, although the growth and feeding performance of the groups were not statistically affected by the NP stimuli. In the early co-feeding stage, larvae of many fish species including sturgeons have a more restricted spectrum of effective taste stimuli, which adversely impacts dry feed intake, adaptability to the new dietary regime, and thereby rising mortality and cannibalism as observed in our results [43–45]. Additionally, the depression of survival rate noticed in SBM groups probably is reflected in immature digestion systems and the detrimental effects of ANFs in plant-based diets on nutrient absorption [10, 46].

The growth performance including final weight, WG, and SGR was slightly higher in fish fed with FM than in the other groups at the end of the NP phase; however, no statistical differences were detected among the treatments. These findings may stem from the short duration of NP, levels of dietary plant protein, and physiological condition of fish. Perera and Yúfera [47] found that after 14 days of SBM feeding, gilthead sea bream (*Sparus aurata*) larvae showed reduced growth, whereas those fed the same diet for 10 days showed normal growth. This explains that the timing of the nutritional stimuli has a determinative role in the pattern of metabolic functions, and fish species differentially respond to the stimuli at different periods of their lifespan. Apart from time frame, many studies demonstrated that nutritional stimulus, programming method, age, weight, or species are the main factors affecting NP efficiency [20, 48, 49].

According to the present study in phase 2, the growth performance in fish fed with a SBM-based diet during the early stage was similar to fish fed with a fish meal-based diet throughout the early stage. This result was in line with Kemski et al. [49], in which yellow perch (*Perca flavescens*) were fed a common diet while exposed to a diet that FM replaced with 70% SBM during the programming phase. However, another research revealed that the growth indices of Atlantic salmon fed a plant-based diet reduced at the end of the programming phase and continued during the common phase, which was most likely due to lower acceptance and feed utilization [50]. These findings revel that various fish species response to feeding strategies in different ways. In phase 2, the water temperature was higher than the phase 1 and held at an average of 25.30 ± 0.34°C, which was optimal for sturgeon growth [51], which corresponded with the higher range of FER in this phase compared to those in phase 1. Kemski et al. [49] also attributed the higher feed efficiency of yellow perch fed 70% SBM, while the water temperature was increased. It has been assumed that optimal rearing temperature allowed juvenile sterlet to continue somatic growth throughout this phase.

In phase 3, our results revealed that juvenile sterlet sturgeon has an appropriate capacity for SBM-based diets when previously experienced during the larval stage, and the feeding utilization was not negatively influenced by different levels of SBM replacing FM (15–45%). In this respect, the study by Clarkson et al. [50] exhibited a higher feeding efficiency and growth rate in Atlantic salmon at the end of the challenge phase which nutritional programmed with a vegetable-based diet containing soy protein concentrate and gluten during the first exogenous feeding (three weeks). Patula et al. [52] also showed that NP with dietary plant protein can imprint the zebrafish to improve dietary SBM utilization at a later stage for better growth. Moreover, the mortality rate was not affected by changing the SBM level or nutritional intervention. Clarkson et al. [50] found no difference in survival rate between fish during the challenge phase, but fish exposed to the plant-based diets had a significantly higher growth rate than the FM group. These results confirmed the effects of nutritional imprinting by plant diet during the early life stage of fish species on improving juvenile feeding and growth rate [53]. Conversely, Perera and Yufera [47] noticed that the growth performance and metabolism of juvenile zebrafish (*Danio rerio*) were not affected by early NP with the SBM-based diet. Moreover, there are some specific compounds released in the water from plant-based diets that can provoke an olfactory imprinting, responsible for higher intakes later in life, as shown in rainbow trout after early exposure to a diet containing a blend of plant protein resources [19]. In sturgeon, olfaction and gustation are believed to be important in imprinting feeding behavior [43]. In our study, an increase in the growth rate of juvenile fish fed with SBM-based diets than FM is probably related to larval olfaction and taste history. Adaptation of fish to a plant-based diet after larval conditioning demonstrated that there is a degree of memory to environmental triggers such as dietary stimulus. Development of flavor memory and stimulation of olfactory senses in rainbow trout experienced with SBM at the onset of feeding regulate the appetite related to these diets was documented by Balasubramanian et al. [54]. Furthermore, transcriptional and physiological changes during early stages significantly enhance fish tolerance to the different factors and innovative feed formulation.

Present results indicated that body chemical composition was not influenced by the substitution of FM with different levels of SBM. A study on yellow perch has shown little to no effect of SBM on tissue composition [55]. Differences in the body compositions of fish can be attributed to age, size, water quality, season, geographic region, etc. [55]; however, the main influential factors are diet quantity and quality [56]. In some studies, replacing FM with plant ingredients highly affects lipogenic pathways, ATP production [57], and fatty acid deficiency [58]. Lipid deposition in the liver of fish fed a diet containing higher levels of SBM might lead to an increase in HSI values. However, in our study, the lipid content of the whole fish body in the entire feeding trial was at the same levels, which means diet composition did not adversely impact the whole body composition.

FM group showed the highest HUFA content which was slightly higher than SBM15, but not significantly different. Plant-based diets are poor in eicosapentaenoic acid (EPA) and docosahexaenoic acid (DHA), so with increasing SBM, the dietary EPA and DHA decreased significantly. In the study by Matani Bour et al. [59], as SBM inclusion was increased in the diet of beluga (*Huso huso*), the concentration of n-6 fatty acids in the muscle also increased, while n-3 fatty acids decreased. These changes are likely associated with the high levels of linolenic acid in the diet when the amount of SBM was high (75%), as suggested in a study by Hosseini et al. [60]. The early nutritional stimuli had no major effect on both EPA and DHA, which have great physiological importance in cell membrane composition. This result may reflect the metabolic adaptation in programmed fish.

Growth hormone (GH) triggers many of the growth-promoting actions by binding to GH receptors and stimulating the synthesis and secretion of insulin-like growth factor-I (IGF-I) from the hepatic tissue [15]. Apart from growth, the GH/IGF-I axis in fish is involved in other physiological and metabolic processes, including osmoregulation, reproduction, and more importantly, nutrition [14, 61, 62]. Many studies showed that changes in GH can result from diet variations [63, 64].

In the present study, fish fed different diets exhibited varied mRNA levels of the *GH* gene; however, the growth performance was not significant among the groups. This result probably arises from multiple inhibitors that regulate fish neuroendocrine and constrain the release of GHs [65]. According to our findings, in phase 1, the mRNA level of *GH* and *IGF-I* was significantly up-regulated in the SBM45 group. Martins et al. [66] documented the crucial role of *IGF-I* and *GH* in repairing the damaged tissue, which is probably related to the deleterious effects of plant-based diets on hepatic tissues in fish fed higher levels of SBM. Further research on histological parameters and oxidative responses may explain the effects of feeding with SBM diets on this species’ well-being. In the challenge phase, we observed the increasing levels of growth-related genes expression in experimental groups, which was allied to the growth performance. This observation is probably due to the effects of nutritional history on the adaptation of tissues and cells to the stimuli. Kumar et al. [67] found an up-regulation of hepatic *IGF-I* transcript in fish fed with a plant-based diet at early life stage, which is in agreement with our findings.

Ghrelin has been identified as the endogenous ligand for the growth hormone and a novel peptide with a potential role in modulating appetite [68, 69]. Our study showed an increasing trend in levels of SBM in diets and mRNA expression of *Ghrelin* at the programming phase; however, in phase 3, the expression levels were highly up-regulated in the SBM45 group. These data also have a consistent correlation with the expression level of the *GH* gene. Studies demonstrated that this hormone has a stimulatory effect on food intake, and growth hormone release. Chen et al. [70] expressed that ghrelin played a pivotal role in promoting the food intake of Siberian sturgeon. Ramezani et al. [71] also observed that the *ghrelin* gene expression was up-regulated in the stomach of Siberian sturgeon when the FI value increased, although no connection was observed between FI and *Ghrelin* levels in the present research. In contrast, some evidence indicates that lower *ghrelin* gene expression level in the gut is possibly related to satiation in fish. Kwasek et al. [53] found that *ghrelin* gene expression in the zebrafish gut decreased in programmed groups. These contradictory results may suggest that the functions of *ghrelin* are species-specific in fish [72].

## Conclusions

To the best of our knowledge, it was the first time to evaluate the feasibility of early NP with plant-based diets on sturgeon, although most of related studies have focused on teleosts. According to the results, sterlet can efficiently utilize SBM-based diets when previously exposed to the same feed during the first exogenous feeding. Therefore, replacing FM with SBM at the level of 45% in the sterlet sturgeon larval feed not only had no deleterious effects on growth performance and body composition but also enhanced mechanisms involved in adaptation to plant-based diets in juveniles. Due to the potential of sterlet sturgeon to be programmed to plant-based diets, further studies are required to evaluate higher levels of plant ingredient inclusion in sturgeon feeding and extend the experimental duration to determine metabolic and physiological mechanisms in the future generations.
